# Deleting the ribosomal prolyl hydroxylase OGFOD1 protects mice against diet-induced obesity and insulin resistance

**DOI:** 10.1371/journal.pone.0304761

**Published:** 2024-06-06

**Authors:** Rebeca Rodriguez, Michael Harris, Leslie M. Kennedy

**Affiliations:** 1 National Heart Lung Blood Institute, National Institutes of Health, Bethesda, MD, United States of America; 2 Department of Physiology, Brody School of Medicine, East Carolina University, Greenville, NC, United States of America; Max Delbruck Centrum fur Molekulare Medizin Berlin Buch, GERMANY

## Abstract

Type 2 diabetes predisposes patients to heart disease, which is the primary cause of death across the globe. Type 2 diabetes often accompanies obesity and is defined by insulin resistance and abnormal glucose handling. Insulin resistance impairs glucose uptake and results in hyperglycemia, which damages tissues such as kidneys, liver, and heart. 2-oxoglutarate (2-OG)- and iron-dependent oxygenases (2-OGDOs), a family of enzymes regulating various aspects of cellular physiology, have been studied for their role in obesity and diet-induced insulin resistance. However, nothing is known of the 2-OGDO family member 2-oxoglutarate and iron-dependent prolyl hydroxylase domain containing protein 1 (OGFOD1) in this setting. OGFOD1 deletion leads to protection in cardiac ischemia-reperfusion injury and cardiac hypertrophy, which are two cardiac events that can lead to heart failure. Considering the remarkable correlation between heart disease and diabetes, the cardioprotection observed in OGFOD1-knockout mice led us to challenge these knockouts with high-fat diet. Wildtype mice fed a high-fat diet developed diet-induced obesity, insulin resistance, and glucose intolerance, but OGFOD1 knockout mice fed this same diet were resistant to diet-induced obesity and insulin resistance. These results support OGFOD1 down-regulation as a strategy for preventing obesity and insulin handling defects.

## Introduction

It is estimated that by 2035, nearly 600 million people worldwide will have diabetes.[1] Type 2 diabetes (T2D), which makes up the majority of diabetes diagnoses,[2, 3] is strongly associated with obesity.[4] The major contributing factors in obesity are diet and comorbidities. As societies have increased their consumption of saturated fat and processed foods, studies have increasingly focused on the links between this Westernized diet and disease. An overwhelming percentage of patients with T2D succumb to cardiovascular disease, with the primary culprits being ischemic heart disease, congestive heart failure, and stroke.[5–8] Despite early detection methods, and treatment strategies that have helped mitigate clinical presentations in patients with T2D, additional work is needed to identify key players functioning in diet-induced pathologies, and the potential for these players to serve as therapeutic targets for improving patient outcomes.

2-oxoglutarate (2-OG)- and iron-dependent oxygenases (2-OGDOs) comprise a large family of enzymes relying on 2-OG and oxygen as co-substrates, and non-heme iron as a cofactor to catalyze hydroxylation of target proteins.[9, 10] 2-OGDOs were initially studied for their roles in modifying collagen,[11] however, they modify a diversity of targets including chromatin, transcription factors, and ribosomal proteins. When down-regulated, the hypoxia-inducible factor prolyl hydroxylase domain enzymes (HIF-PHDs) have been implicated in protection against diet-induced obesity,[12, 13] but there are essential 2-OGDOs whose functions within this context have not yet been investigated. Members of this family have been described as “metabolic sensors” based on their reliance on oxygen, iron, and the TCA intermediate 2-OG.[14] Tpa1p, the *Saccharomyces cerevisiae* homolog of OGFOD1, regulates mRNA transcript stability, poly(A) tail length, and termination efficiency in translation;[15] while the yeast *Schizosaccharomyces pombe* homolog Ofd1 functions in the sterol response pathway by regulating the stability of sterol regulatory element binding protein, Sre1.[16] Mammalian OGFOD1 has diverse roles in cell physiology including polysome dynamics, stress granule formation, eIF2-mediated translation regulation, protein turnover, and cell proliferation.[17–19]

To understand how changes in ribosomal prolyl hydroxylation impacts physiology, we deleted the ribosomal hydroxylase 2-oxoglutarate and iron-dependent prolyl hydroxylase domain containing protein 1 (OGFOD1) in mice. OGFOD1 has been shown to hydroxylate ribosomal protein s23 (RPS23), and regulate synthesis of proteins functioning in metabolic regulation, splicing, and cell differentiation.[20, 21] We challenged OGFOD1-KO mice with 2 risk factors for heart failure—ischemia-reperfusion injury (IRI) to model acute myocardial infarction, and hypertrophy to model more chronic injury. OGFOD1 deletion led to protection against both IRI,[22] and hypertrophy as compared to their wildtype (WT) counterparts.[23]

There is a well-established link between heart disease and insulin resistance in patients.[6, 24–27] Based on our published evidence that OGFOD1 plays a role in protection against both acute and chronic cardiac pathologies, we tested whether OGFOD1 deletion impacted susceptibility to obesity, insulin resistance, and reduced glucose tolerance that develop following high-fat diet feeding. In this study, we challenged OGFOD1-KO mice with high-fat diet (HFD) feeding, and assessed their weight gain, insulin sensitivity, glucose tolerance, and cardiac function. OGFOD1-KO mice were protected from HFD-induced weight gain over the course of the study, and weighed significantly less than their HFD-fed OGFOD1-WT (WT) counterparts at the end of the study. Upon closer examination, we found that KO mice were leaner than WT mice when fed the HFD, and also trended toward a leaner phenotype when fed a standard diet (SD). Glucose tolerance was largely comparable between WT and KO mice fed an HFD, but insulin sensitivity was significantly better in HFD-fed KO mice than HFD-fed WT mice. Additionally, KO mice maintained AKT activity between SD and HFD. Overall, these results indicate OGFOD1-KO mice are protected from HFD-induced obesity and insulin insensitivity.

## Methods

### Mice

Embryonic stem cell clones with a targeted *Ogfod1* knockout allele (*Ogfod1*^*tm1a(KOMP)Wtsi*^) were retrieved from the European Conditional Mouse Mutagenesis Program. The targeted allele contained a lacZ/neomycin antibiotic resistance cassette flanked by FRT sites, and an early region of the *Ogfod1* genomic sequence flanked by loxP sites. Embryonic stem cells were ultimately used to produce chimeric males, which were bred to C57BL/6 mice to produce progeny carrying the targeted allele. Flp-deleter mice were then used to eliminate the antibiotic resistance cassette and in subsequent breeding, Pgk-Cre mice were used to induce *Ogfod1* deletion, as previously described.[19] *Ogfod1-KO* mice were maintained on a Taconic C57/BL6N background. All animal studies were performed in a manner consistent with the recommendations established by the Guide for the Care and Use of Laboratory Animals (National Institutes of Health), and all animal protocols were approved by the National Heart, Lung and Blood Institute’s Animal Care and Use Committee. Age-matched OGFOD1-WT and OGFOD1-KO male mice aged 3–5 months were used for this study. For the High-fat Diet (HFD) study, mice were fed a standard diet (NIH-31) or a high-fat diet (Research Diets Inc. #D12492) *ad libitum* for 11 weeks. In accordance with approved guidelines, mice were euthanized by intraperitoneal injection of 125 mg/kg pentobarbital sodium salt (Sigma #P3761). Euthanasia was confirmed by failure of the animal to respond to firm toe pinch.

### Glucose and insulin tolerance tests

In week 9 of the HFD study, mice were fasted for 6 hours with free access to water. Mice were then given an intraperitoneal injection of glucose (1.5 mg/gram body weight) or insulin (0.5 milliunits of human rapid acting insulin/gram body weight). Blood glucose was measured at 15, 30, 60, 90 and 120 minutes after glucose or insulin injection. Fasting blood glucose was measured using a glucometer, and the glucose tolerance test (GTT) and insulin tolerance test (ITT) were done one week apart.

### Fat and lean mass measurements

Echo-magnetic resonance imaging (EchoMRI) was used to estimate lean and fat masses in un-anesthetized mice via nuclear magnetic resonance imaging in the final week (week 11) of the HFD study. Average lean and fat masses were calculated for each mouse from two scans.

### Blood insulin measurements

Mice were fasted for 6 hours prior to blood collection. Whole blood was allowed to clot by incubating at ambient temperature for 15 minutes. Blood was centrifuged at 2000 g for 20 minutes at 4°C. The supernatant, which is the serum, was then used in the Ultra Sensitive Mouse Insulin ELISA kit (Crystal Chem #90080).

### β-hydroxybutyrate measurements

Gastrocnemius muscles were dissected from fed mice and flash-frozen. Tissues were ground to a fine powder liquid nitrogen, and assayed for β–hydroxybutyrate using Promega’s BHB-Glo™ (Ketone Body) Assay Kit (# JE9500) following the manufacturer’s protocol.

### Western blot

Mouse gastrocnemius muscle was homogenized in Radioimmunoprecipitation assay buffer (ThermoFisher #89901) amended with protease inhibitors (Roche #04-693-159-001). This tissue was homogenized by mechanical disruption on the Precellys® 24 with Cryolys (Bertin Technologies) tissue homogenizer chilled to 4°C. For AKT, pAKT, and GAPDH, protein lysates were combined with Laemmli buffer and separated by Tris-glycine SDS-PAGE. For BDH1 and HSC70, protein lysates were combined with NuPage LDS buffer and separated by Bis-Tris SDS-PAGE. Each gel was transferred to 0.45 um nitrocellulose membrane, blocked in 5% milk, and probed with primary and secondary antibodies to determine specific protein abundances. Antibodies can be found in S1 Table. All full blots can be found in S1 Fig.

### Statistics

A Mann-Whitney test was used to compare 2 groups. Two-way Analysis of Variance (ANOVA) was with post-hoc multiple comparisons tests used to compare 2 groups with 2 sub-types in each of the 2 main groups (e.g. WT and KO groups with diet sub-types). A P-value less than 0.05 was considered significant. All raw data can be found in S2 Table.

## Results

### Ablating OGFOD1 protects against high-fat diet-induced weight gain

**Fig 1:**
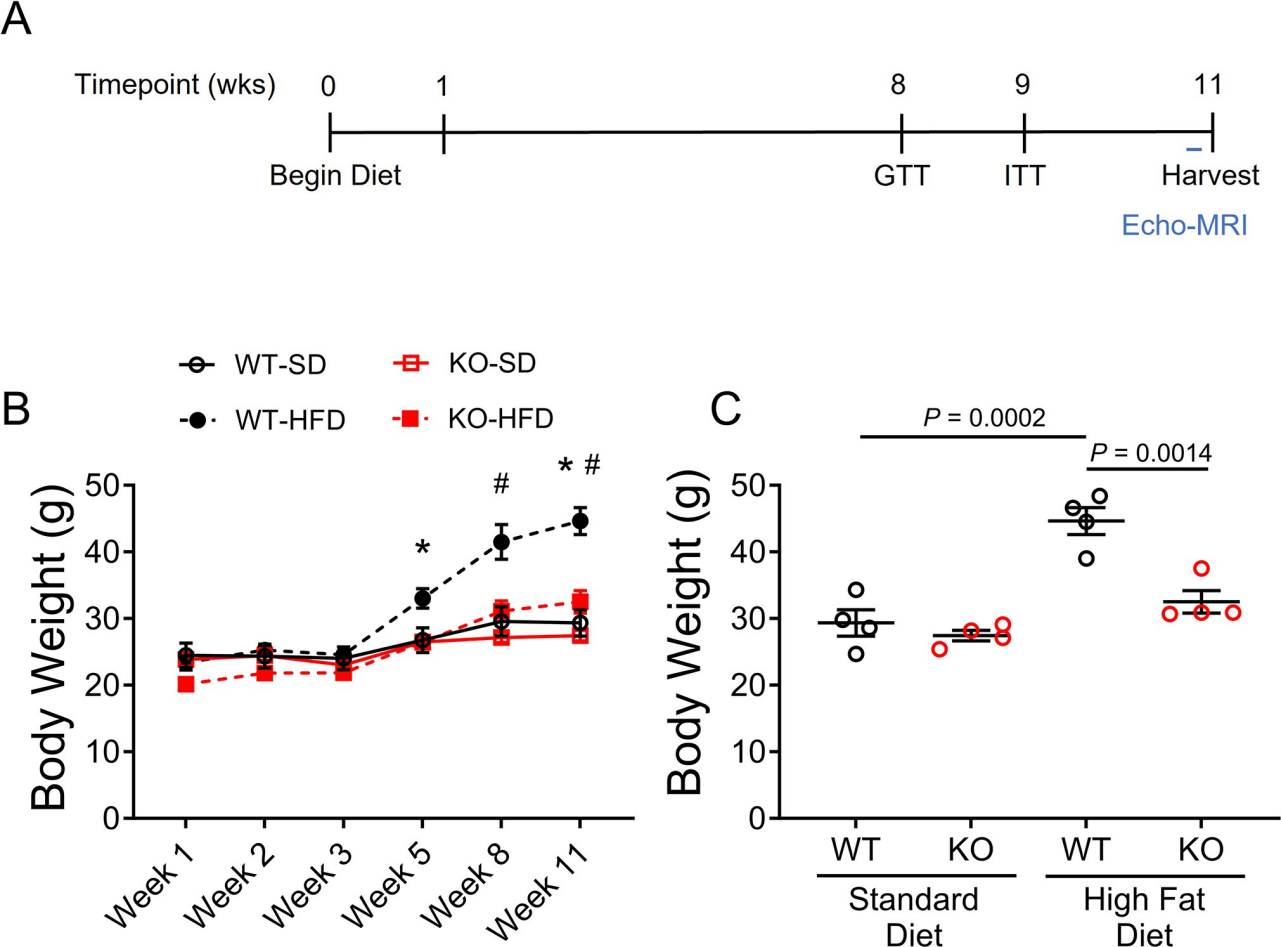
OGFOD1 loss protects mice from high-fat diet-induced weight gain. **A**. Schematic showing the timeline of key events over the course of the high fat diet study. **B**. Weight gain over the course of the high-fat diet study in mice fed a standard diet (SD) or high-fat diet (HFD). **C**. Total body weights at study-end in WT and KO mice fed a standard or high-fat diet. Data shown as mean ± standard error. Repeated measures ANOVA with Geisser-greenhouse correction and Tukey multiple comparisons test was used for B, *P* = 0.0112. Two-way ANOVA with post-hoc multiple comparisons test was used to compare groups in C, *P* < 0.0001. ^#,^*P < 0.05. In panel B, asterisk (*) refers to significant differences between WT-HFD and KO-HFD, hash tag (#) refers to significant differences between WT-SD and WT-HFD. For panel C, significant *P* values are shown.

To understand the relationships between OGFOD1 and systemic metabolism, we subjected WT and *Ogfod1*-knockout (KO) mice to a standard diet (SD) or a high-fat diet (HFD) *ad libitum* for 11 weeks (Fig 1A). In tracking weight gain over the course of the study, all mice started at similar weights, and only WT mice fed a HFD gained significantly more weight over the course of the study than the remaining groups (Fig 1B). KO mice gained weight at a rate comparative to the WT and KO mice fed SD. At the end of this feeding regimen, HFD-fed KO mice weighed 25% less than HFD-fed WT mice (40.7 ± 4.4 g in WT-HFD v. 30.5 ± 2.7 g in KO-HFD, *P* < 0.005), while WT and KO mice fed a SD showed comparable weights (Fig 1C). Based on these results, OGFOD1 deletion protects mice against HFD-induced weight gain.

### Ablating OGFOD1 protects against high-fat diet-induced accumulation of fat

**Fig 2:**
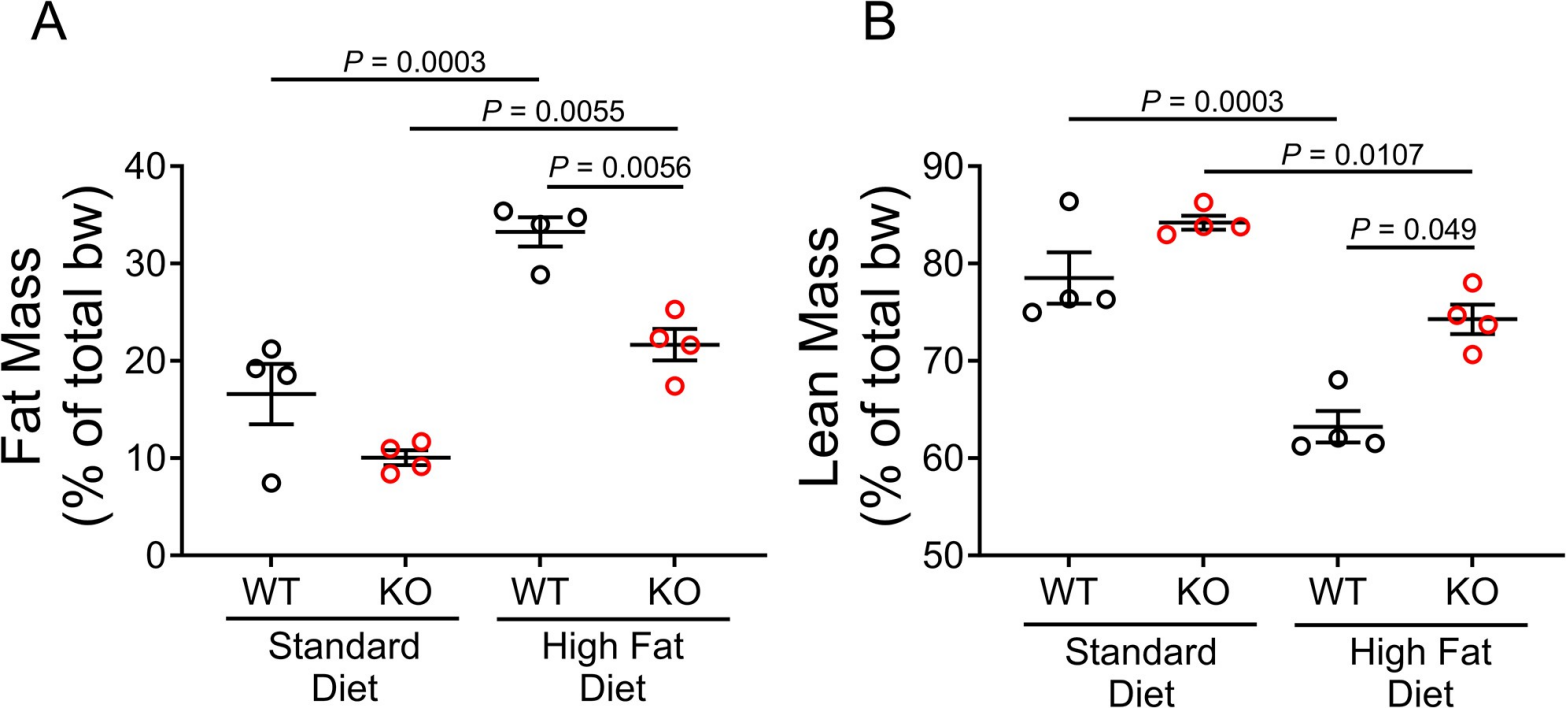
OGFOD1 loss protects mice from high-fat diet-induced fat accumulation. **A**. Fat mass as a percentage of total body weight. **B**. Lean mass as a percentage of total body weight. Data shown as mean ± standard error. Two-way ANOVA with post-hoc multiple comparisons test was used to compare more than 2 groups. Significant *P* values are shown.

We were interested in identifying whether KO mice fed a HFD had different overall fat mass compared to WT mice fed a HFD, so we assessed fat and lean masses using echo-MRI. KO mice and WT mice fed a SD showed comparable fat mass percentages (16.6 ± 6.2% in WT-SD v. 10.1 ± 1.5% in KO-SD, *P* = 0.133). When we examined mice fed a HFD, we found that HFD-fed KO mice showed a significantly lower fat mass percentage than HFD-fed WT mice (33.2 ± 3.0% in WT-HFD v. 21.7 ± 3.2% in KO-HFD, *P* = 0.006) (Fig 2A). As expected, lean mass complemented fat mass, with KO mice showing no significant difference from WT mice when fed the SD (78.5 ± 5.3% in WT-SD v. 84.2 ± 1.4% in KO-SD, *P* = 0.229). However, with HFD feeding, KO mice were leaner, showing a 17.5% higher percentage of lean mass than WT mice (63.2 ± 3.2% in WT-HFD v. 74.3 ± 3.0% in KO-HFD, *P* = 0.005) (Fig 2B). Overall, fat mass was a lower percentage of overall body weight in KO mice fed a HFD than in WT mice fed a HFD, and KO mice fed a HFD were leaner than WT mice fed a HFD. Together, this indicates KO mice are resistant to HFD-induced fat accumulation, as compared to WT mice.

### Ablating OGFOD1 protects against high-fat diet-induced insulin resistance

**Fig 3:**
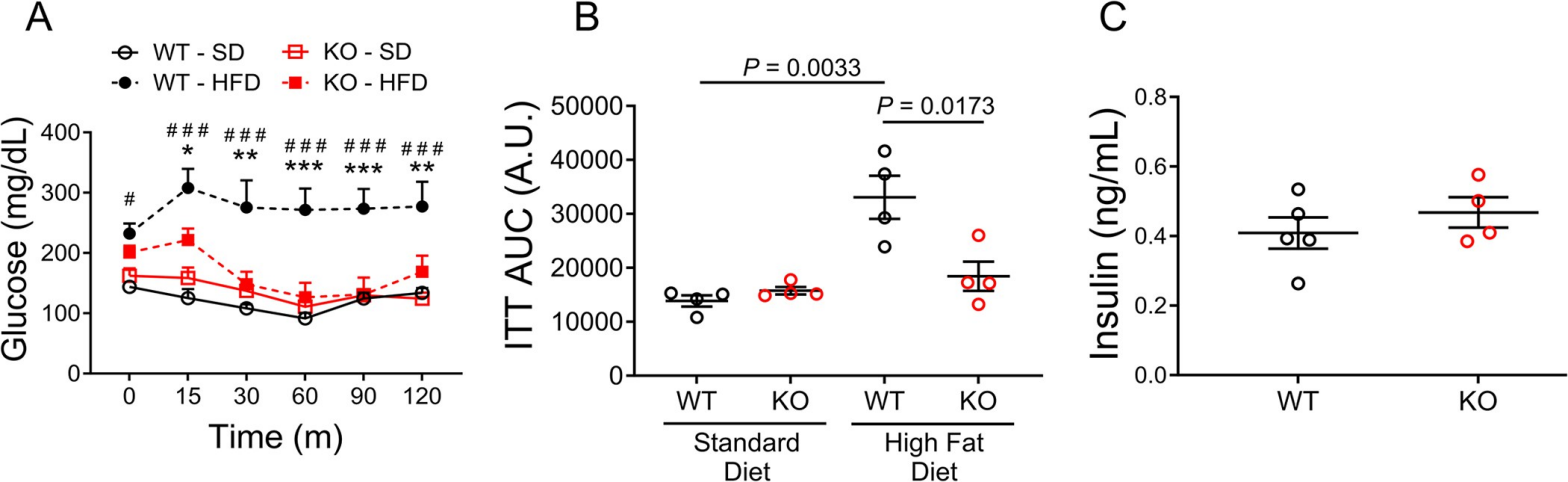
OGFOD1 loss protects mice from high-fat diet-induced insulin resistance. **A**. Insulin tolerance test (ITT) results are shown based on blood collected for measurement of glucose levels at several timepoints over a 2-hour timeframe. **B**. Area under the curve (AUC) for the ITT. **C**. Fasting circulating insulin levels. Data shown as mean ± standard error. Repeated measures ANOVA and Tukey multiple comparisons test was used to compare groups in panel A, *P* = 0.0005. Two-way ANOVA with Tukey multiple comparisons test was used to compare more than 2 groups in panel B, *P* = 0.0028. Mann-Whitney test was used to compare 2 groups in panel C. ^#,^*P < 0.05; **P < 0.005; ^###,^***P < 0.0005. In panel A, asterisks (*) refer to significant differences between WT-HFD and KO-HFD, hash tags (#) refer to significant differences between WT-SD and WT-HFD.

Diets high in fat can impact insulin signaling and glucose uptake as lipid accumulation becomes toxic to tissues. Because KO mice failed to gain weight similarly to their WT counterparts, we next determined the capacity for increased leanness to be accompanied by improved insulin sensitivity in KO mice. To determine this, an insulin tolerance test (ITT) was performed. For the ITT, the mice were fasted for 6 hours, and fasted blood glucose levels were measured. Next, the mice were injected with insulin, and blood was collected at multiple time points over 2-hours to assess blood glucose levels. WT and KO mice fed a SD showed similar rates of insulin-stimulated glucose clearance (Fig 3A and 3B), and similar levels of basal insulin (Fig 3C). However, when the mice were fed an HFD, KO mice showed significant improvements in glucose sensitivity over their WT counterparts (Fig 3A and 3B). As expected, given the protection from weight gain, KO mice displayed glucose tolerance comparable to the SD-fed mice.

### Ablating OGFOD1 slightly improves glucose-stimulated glucose uptake

**Fig 4:**
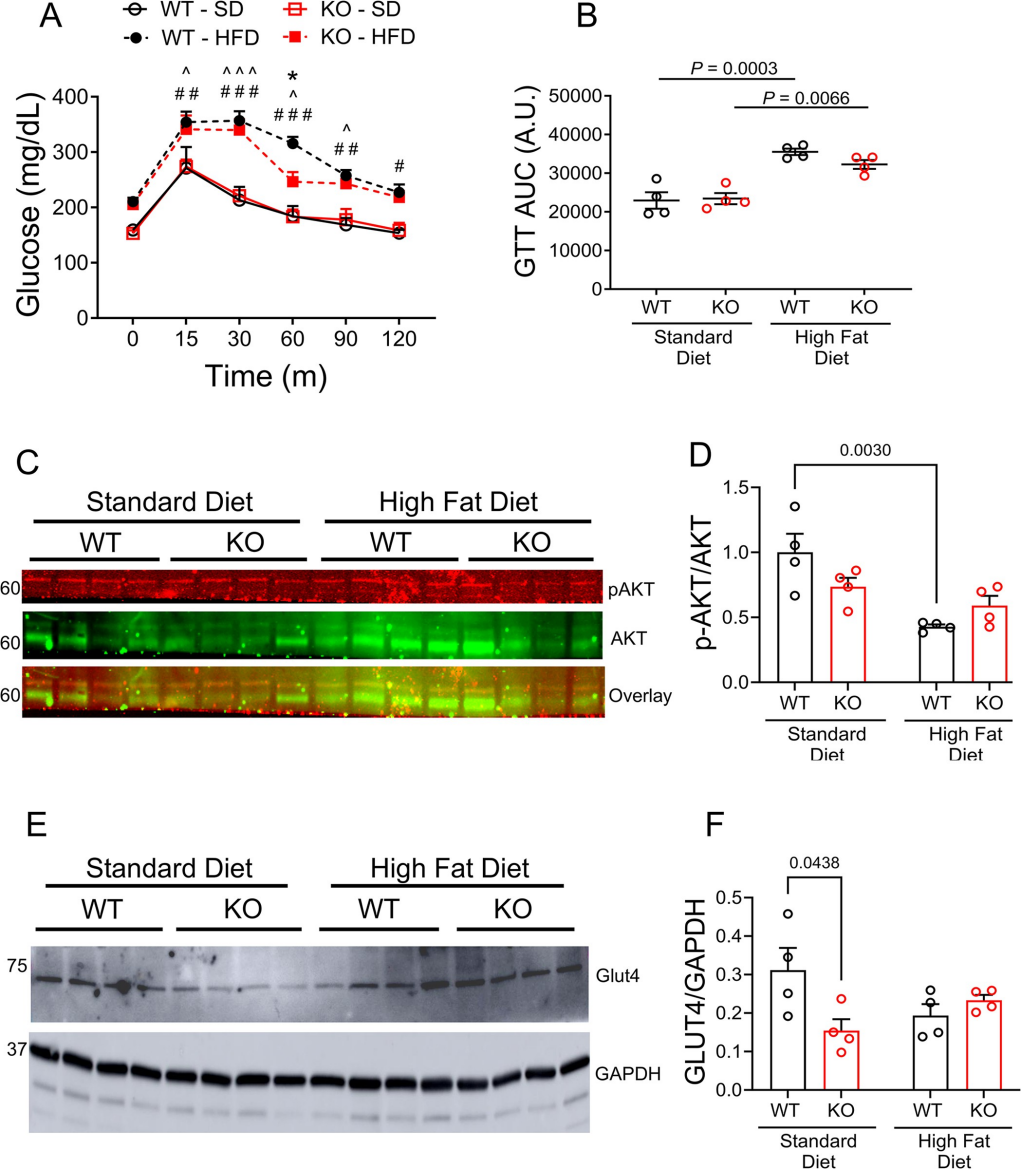
OGFOD1 loss has little impact on high-fat diet-mediated glucose tolerance. **A.** Glucose tolerance test (GTT) results are shown based on blood collected for measurement of glucose levels at several timepoints over a 2-hour timeframe. **B.** Area under the curve (AUC) for the GTT. **C.** Phosphorylated and total AKT protein levels in skeletal muscle protein isolates. **D.** Densitometry results for the abundance results shown in panel C. **E.** Glut4 protein levels in skeletal muscle protein isolates. **F.** Densitometry results for the abundance results shown in panel E. Data shown as mean ± standard error. Repeated measures ANOVA and Tukey multiple comparisons test was used to compare groups in panel A, *P* < 0.0001. Two-way ANOVA was used to compare more than 2 groups in panels B, D, F. ^#,^,*^P < 0.05, ^##^*P* < 0.005, ^###,^^^^*P* < 0.0005. Hash tags (#) refer to significant differences between WT-SD and WT-HFD, carats (^) refer to significant differences between KO-SD and KO-HFD, and asterisks (*) refer to significant differences between WT-HFD and KO-HFD.

HFD can be associated with both insulin resistance and a decline in glucose sensitivity. In light of our findings that KO mice were protected from HFD-induced insulin resistance, we investigated glucose tolerance. To accomplish this, mice were fasted for 6 hours, blood was collected and used to measure fasting blood glucose levels. For the glucose tolerance test (GTT), fasted mice were injected with glucose, and blood was collected at multiple time points over a 2-hour time frame to assess blood glucose levels. WT and KO mice showed similar rates of glucose-stimulated glucose clearance when fed a SD (Fig 4A and 4B). When fed a HFD diet, WT and KO glucose clearance was comparable up until 60 minutes after glucose injection, where KO mice fed a HFD no longer had blood glucose levels that were significantly higher than KO mice fed a SD. These results indicate OGFOD1 deletion slightly improves the response to glucose-stimulated glucose uptake.

OGFOD1-KO mice were protected in insulin-stimulated glucose uptake, but behaved similarly to WT mice in glucose-stimulated glucose uptake. Glucose stimulates pancreatic beta cells to release insulin, which in turn binds insulin receptors and activates a signal transduction cascade involving a series of events which include AKT phosphorylation and glucose uptake. To identify molecular differences that may contribute to the OGFOD1-KO phenotype, we focused initially on AKT signaling, which can be activated downstream of insulin binding the insulin receptor. AKT phosphorylation was down-regulated by 57.2% upon HFD feeding in WT mice (*P* = 0.0030), however OGFOD1-KO mice showed no difference in signaling from SD to HFD feeding (Fig 4C and 4D). These results are consistent with OGFOD1-KO mice retaining insulin-stimulated glucose uptake, even after HFD feeding. Once insulin stimulates AKT signaling, a signal cascade leads to Glut4 translocating to the cell membrane to facilitate glucose uptake. In mice fed a SD, Glut4 expression was lower in KO mice compared to WT mice. Glut4 was unchanged between WT and KO mice fed HFD (Fig 4E and 4F), and from SD to HFD for the WT and KO groups. These results show reduced insulin signaling from standard to high-fat-diet feeding in WT mice, while KO mice maintain their insulin signaling activity between diets.

### OGFOD1 deletion leads to a decline in the ketone body β-hydroxybutyrate

**Fig 5:**
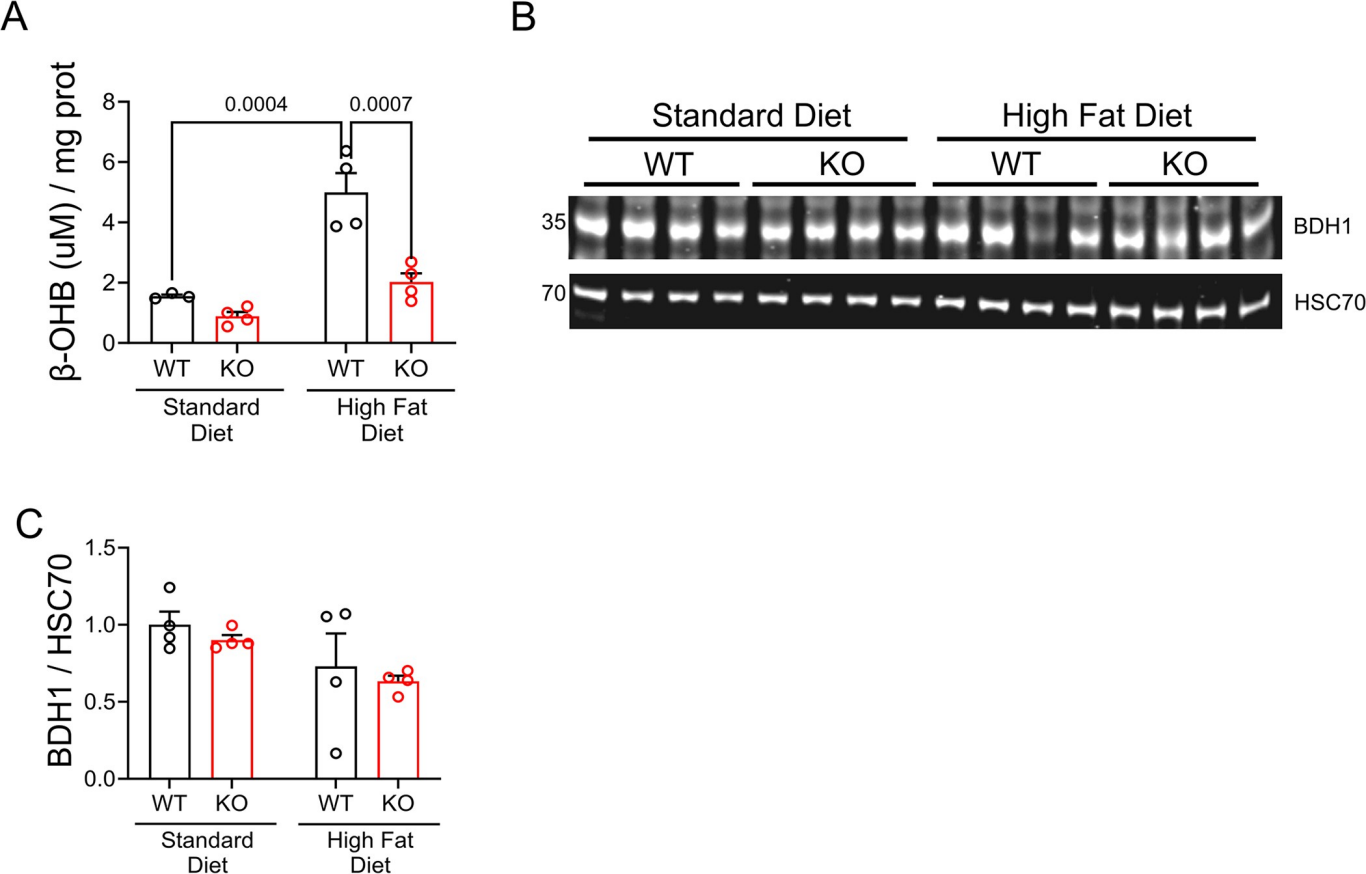
β–hydroxybutyrate levels are lower in KO than WT murine skeletal muscle from mice fed a high-fat diet. **A**. β–hydroxybutyrate (β-OHB) levels measured in lysates from gastrocnemius muscles. **B**. β–hydroxybutyrate dehydrogenase 1 (BDH1) protein levels in lysates from gastrocnemius muscles. **C**. Densitometry results for the abundances shown in panel B. Data shown as mean ± standard error. Two-way ANOVA was used to compare more than 2 groups in A and C.

In mice fed a standard diet, OGFOD1-KO cardiac tissue showed a significant 60% decline in β-hydroxybutyrate (β-OHB) levels,[28] with no change in circulating β-OHB in fasted mice (data not shown). This indicated the potential for OGFOD1 deletion to lead to enhance ketone oxidation either by increasing the levels or activity of ketone oxidation enzymes. We measured β-OHB levels in gastrocnemius lysates from the hindlimbs of mice fed a SD or HFD. Interestingly, HFD-fed KO mice had 60% lower β-OHB levels than HFD-fed WT mice (Fig 5A). In determining whether this change could be attributed to a change in β–hydroxybutyrate dehydrogenase 1 (BDH1) protein levels, a key enzyme in oxidizing β-OHB, we measured BDH1 levels and found no significant differences among the groups (Fig 5B and 5C). These results indicate OGFOD1 deletion impacts the levels of the ketone body β-OHB, which suggests the potential for ketone body utilization to be altered in KO mice.

## Discussion

Hypoxia-inducible factor (HIF) prolyl hydroxylase domain enzymes (PHDs) are well-known members of the 2-OGDO family. HIF-PHDs are oxygen-dependent enzymes that are functional under normoxic conditions, but inactive under hypoxic conditions. When active, these enzymes target HIF for proteosomal degradation.[29–32] However, when inactive, such as what occurs under hypoxic conditions, HIF-PHDs cannot target HIF for degradation, therefore HIFα accumulates, heterodimerizes with its β subunit, and functions as a transcription factor for genes such as metabolic enzymes, angiogenic and hematopoietic factors, and growth factors.[33, 34]

Deleting the HIF1α 2-OGDO enzyme PHD2 in mouse cardiomyocytes (PHD2^cardiacKO^) and feeding these mice an HFD led to protection against diet-induced obesity, elevated fasting blood-glucose levels, and cardiac dysfunction.[13] Adipocyte-specific PHD2 deletion led to protection in obesity, glucose tolerance, and insulin sensitivity.[12] Inhibiting PHD activity using JTZ-951 led to protection against weight gain and the immune response elicited following HFD feeding.[35] Based upon the diverse transcriptional targets of HIFs, these non-ribosomal prolyl hydroxylases have been investigated for their role in obesity, cancer, kidney disease, and other illnesses. Therapeutic interventions targeting HIF-PHDs have also been shown to inhibit other 2-OGDO family members, rendering it important to understanding how down-regulating remaining 2-OGDOs relate to HFD affects. The current study is the first to investigate the potential for ribosomal prolyl hydroxylases to function in HFD.

Here, we demonstrate that deleting the ribosomal proline oxygenase OGFOD1 in mice confers a protective benefit when these KO mice are fed an HFD. HFD-fed KO mice fail to gain weight at the same rates as their HFD-fed WT counterparts, and weigh significantly less than WT mice after 11 weeks of HFD feeding. Consistent with this, HFD-fed KO mice had significantly lower fat deposition. HFD feeding can lead to fat accumulating in tissues to a toxic level, which then impacts metabolism. To investigate this, we looked at glucose handling and insulin signaling capabilities. When fed a SD, WT and KO mice responded similarly to glucose or insulin boluses, indicating no basal differences in either glucose handling or insulin signaling upon losing the proline hydroxylase OGFOD1. HFD-fed KO mice injected with insulin, behaved similarly to SD-fed animals, and were protected from HFD-induced insulin resistance. KO mice fed an HFD showed some protection in the glucose tolerance test 60 minutes following glucose injection, where KO mice fed HFD appeared to clear glucose more rapidly from the blood than WT mice fed HFD. Thus, KO mice were protected in insulin-stimulated glucose uptake, but failed to demonstrate full protection in glucose-stimulated glucose uptake despite the SD-fed WT and KO mice having comparable levels of fasting blood insulin. Determining whether KO-HFD and WT-HFD mice maintain comparable fasting levels of circulating insulin is an important future pursuit.

OGFOD1 hydroxylates proline 62 of RPS23. This residue has been shown to be uniquely hydroxylated by OGFOD1; and in the absence of OGFOD1, this residue is no longer hydroxylated.[19, 21] The OGFOD1 target RPS23 resides in the ribosomal decoding center,[36] where mRNA is brought in close proximity to tRNA to ensure proper translation fidelity based on the mRNA codon sequence. Prolyl hydroxylation catalyzed via HIF-PHDs is a well-known mechanism for regulating protein stability. However, OGFOD1 deletion had no impact on the stability of its best described target, RPS23.[21] Rather than impacting its turnover, OGFOD1-mediated prolyl hydroxylation may regulate translation by impacting the RPS23 transcript pool that is selected for synthesis. Phosphorylation on ribosomal protein s6 (Rps6) was the first ribosomal post-translational modification (PTM) identified that could be induced,[37, 38] and is often used as a readout for mechanistic target of rapamycin (mTOR) signaling activity or neuronal activity.[39–41] Interestingly, Rps6 phosphorylation modifies the pool of transcripts that are translated as opposed to blocking global translation.[42] A similar mechanism may apply when RPS23 is no longer hydroxylated.

The protein groups showing the most change with OGFOD1 deletion in mouse cardiac tissue are translational machinery, cytoskeletal architecture, and metabolism.[22] Additionally, OGFOD1-KO mice demonstrated a 60% decline in cardiac β-OHB according to metabolomic data, indicating a potential change in ketone production, release, or utilization. In isolated cardiomyocytes, prolonged treatment with exogenous β-OHB stunted insulin-stimulated glucose uptake and subsequent insulin action.[43] This was supported by a human study showing β-OHB infusion led to a decline in cardiac glucose uptake.[44] Suppressing ketone oxidation by deleting succinyl-CoA:3-ketoacid-CoA transferase (SCOT), a key enzyme in β-OHB oxidation, reversed hyperglycemia in obese mice.[45] Here, we show that KO murine skeletal muscle shows a significant decline in β-OHB, with no change in the β-OHB oxidizing enzyme BDH1. Assessing the activities of BDH1 and other key β-OHB-metabolizing enzymes is an important future direction in establishing whether the decline in skeletal β-OHB occurs due to extra-hepatic enzyme activity. The connection between OGFOD1, changes in skeletal muscle β-OHB levels, and protection in high fat diet, highlight the capacity for OGFOD1 to regulate these metabolic pathways, likely through changes at the translational level. More work is needed to directly link OGFOD1 translational targets to the protection that occurs upon high-fat diet feeding. These studies are an essential component in understanding the benefits of OGFOD1 down-regulation as a strategy for managing obesity and the systemic dysfunction associated with this disease.

## Supporting information

S1 Table Antibodies. (XLSX)

S2 Table Minimal data. (XLSX)

S1 Fig Unedited blots. (PDF)
