# Supplementary material for: Deleting the ribosomal prolyl hydroxylase OGFOD1 protects mice against diet-induced obesity and insulin resistance
Source: PLoS One. 2024 Jun 6;19(6):e0304761. doi: 10.1371/journal.pone.0304761 (PMC11156292; doi:10.1371/journal.pone.0304761)
Supplement: S1 Fig — (PDF) [file pone.0304761.s003.pdf]

A

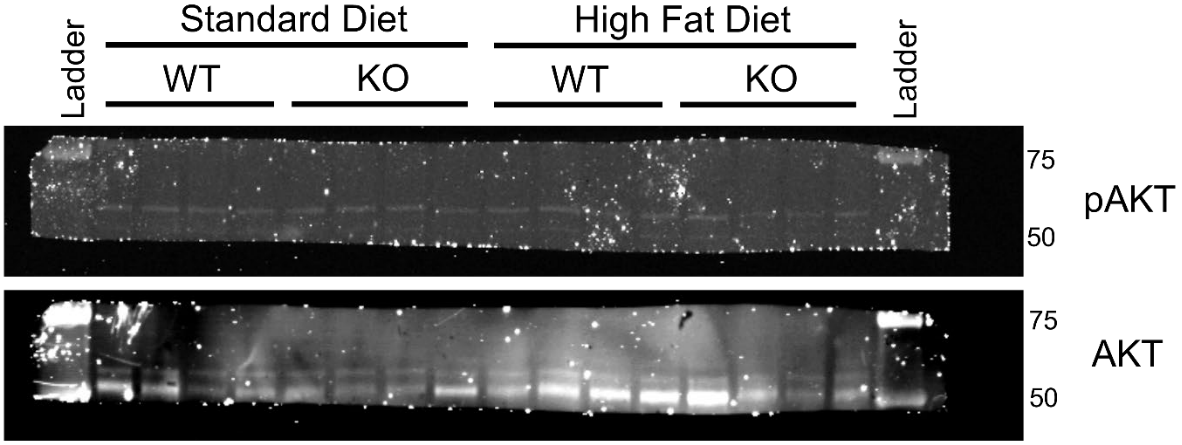
Ladder
Standard Diet
High Fat Diet
WT
KO
WT
KO
Ladder
75
50
pAKT
75
50
AKT

B

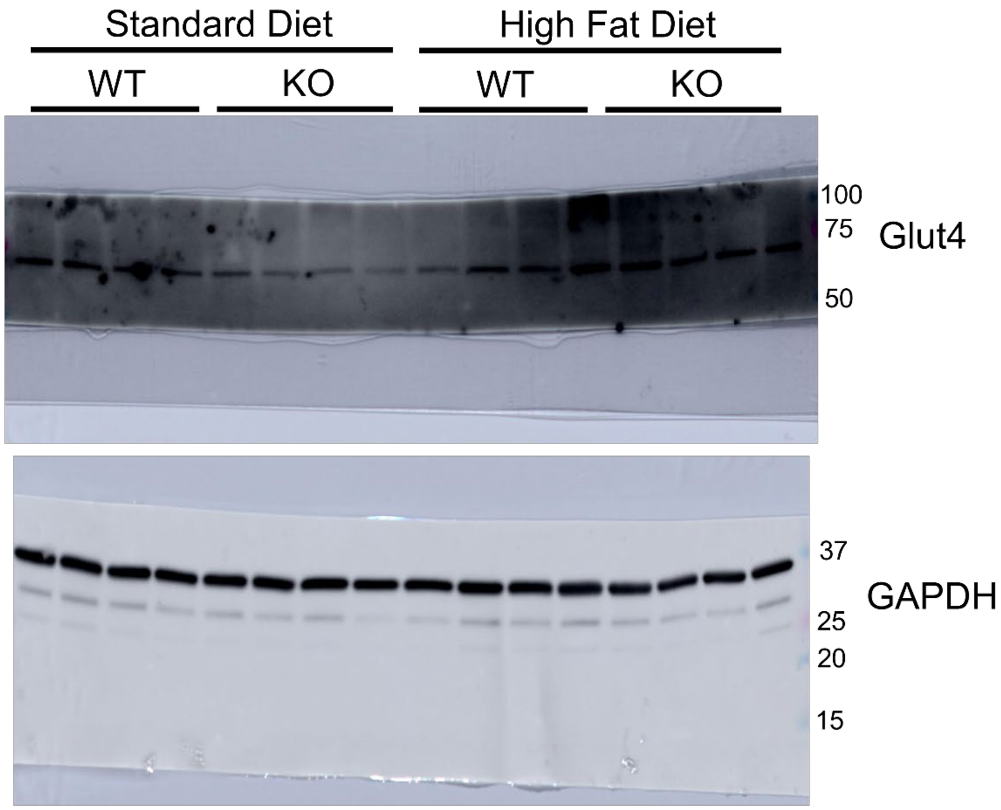
Standard Diet
High Fat Diet
WT
KO
WT
KO
100
75
50
Glut4
37
25
20
15
GAPDH

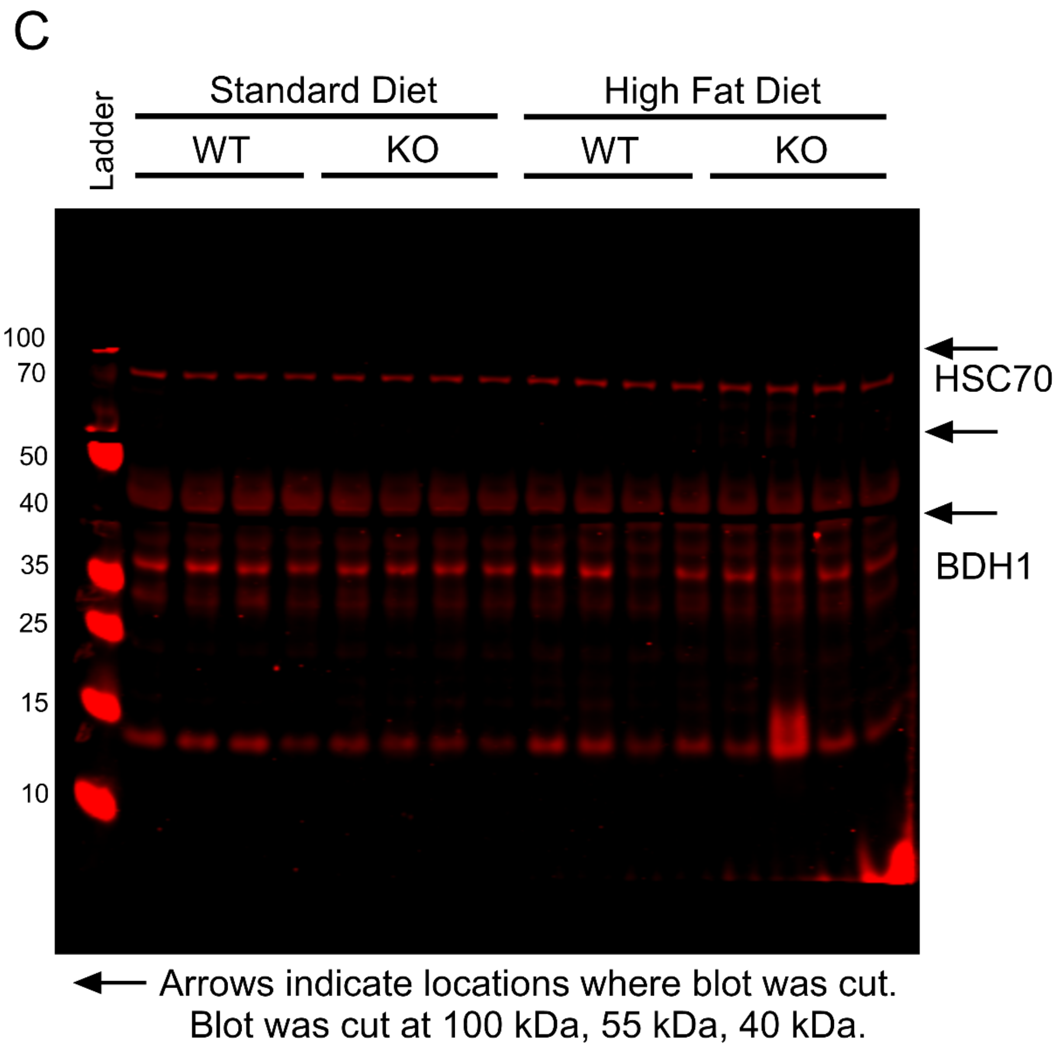

Fig S1. Full blots.

Full blots from manuscript Figure 4C (shown in panel A), Figure 4E (shown in panel B), and Figure 5B (shown in panel C).
